# Exploring the drivers of agricultural wages growth in China: A comprehensive framework utilizing input-output and structural decomposition methods

**DOI:** 10.1371/journal.pone.0299067

**Published:** 2024-03-27

**Authors:** Peijiang Zheng, Yang Li, Yingying Qi

**Affiliations:** 1 School of Economics, Sichuan University, Chengdu, Sichuan, China; 2 School of Economics, Northwest Minzu University, Lanzhou, Gansu, China; Institute for Economic Forecasting, Romanian Academy, ROMANIA

## Abstract

This study explores the factors driving agricultural wages growth in China from 1981 to 2020. We propose a comprehensive framework that combines input-output analysis and structural decomposition analysis to investigate the drivers of agricultural wages growth from four perspectives: supply, demand, industrial linkages, and agricultural support policies. The findings indicate that changes in consumer demand, investment demand, and labor mobility play significant roles in driving the growth of agricultural wages in China. Additionally, agricultural support policies have contributed to an increase in agricultural wages to some extent. However, changes in industrial linkages negatively affect agricultural wages growth. A notable strength of this study lies in the methodology employed, which ensures a comprehensive and systematic analysis encompassing diverse factors rather than a restricted perspective.

## 1. Introduction

It is widely recognized that the real wage of Chinese farmers experienced slow growth in the two decades following China’s reform and opening-up [1]. However, in recent years, the growth rate of Chinese agricultural wages has accelerated [2]. From 1981 to 2000, adjusted for the constant prices in 2010, the average wage of employed persons in Chinese agricultural sector increased by 2.45 times, with an average annual growth rate of 4.82%. In contrast, from 2000 to 2020, Chinese agricultural wages surged by 10.11 times, with an average annual growth rate of 12.27%.

What factors account for this notable disparity in the growth rates of Chinese agricultural wages between the initial and subsequent two decades? More importantly, what were the driving forces behind the rapid growth observed between 2000 and 2020?

For an extended duration, scholars have dedicated their attention to the examination of supply [3–5], and demand [2, 6–8] as pivotal factors for comprehending the dynamics of agricultural wages. Notably, numerous researchers have conducted investigations into the influence of industrial linkages [8–10] and agricultural support policies [11–14] on agricultural wages.

However, the majority of research has predominantly explored the influence of singular or a limited number of factors on agricultural wages, frequently neglecting the incorporation of these factors into a comprehensive analytical framework. In other words, the existing literature is deficient in a systematic research framework for appraising the relative significance of these factors in relation to agricultural wages. To gain a comprehensive understanding of the changes in agricultural wages in China, it is essential to consider not only the supply and demand factors but also the impact of changes in industrial linkages and agricultural support policies. Recognizing this research gap, this study employs both input-output analysis (IOA) and structural decomposition analysis (SDA) methods to systematically analyze the drivers of agricultural wages growth in China.

The main contributions of this study are as follows: (1) This study innovatively integrated supply, demand, industrial linkages, and agricultural support policies into a comprehensive analytical framework. This facilitated a thorough examination of how supply factors influence agricultural wages through labor and product markets and how demand factors and industrial linkages directly and indirectly impact agricultural wages. It also explored how agricultural support policies stimulate agricultural development, thereby affecting agricultural wages. (2) The proposed framework allowed the identification of correlations between these factors and agricultural wages. More importantly, it enabled quantification of the relative contribution of each factor to agricultural wages. (3) Focusing on China as the research subject, this study revealed the driving factors behind agricultural wages growth over the past 40 years. This highlighted the dynamic changes in agricultural wages during the process of economic development and analyzed the corresponding impact of agricultural support policies. Overall, the findings of this study have important policy implications, as they enhance our understanding of the factors influencing agricultural wages and provide insights for policymakers in developing countries.

The remainder of this paper is organized as follows. Section 2 reviews the existing literature. Section 3 describes the methods and data employed in this study. Section 4 presents the results and provides an in-depth analysis of the underlying factors. Section 5 is dedicated to the discussion, and Section 6 summarizes the study.

## 2. Literature review

Numerous scholars have explored the factors influencing agricultural wages growth. These factors can be broadly categorized into four groups: supply, demand, industrial linkages, and agricultural support policies.

### Supply side

Regarding the supply side, previous studies have identified several factors that affect agricultural wages. These factors include rural labor transfer, agricultural labor productivity, agricultural mechanization, and plant biotechnology.

#### Rural labor transfer

China has experienced rapid economic development since the implementation of market-oriented reforms following its reform and opening-up [15]. Furthermore, the Chinese government has gradually relaxed restrictions on rural migration [5, 16, 17], leading to a common trend where farmers seek lucrative employment opportunities in urban areas. Increasing wage rate differentials continue to incentivize rural-urban migration [2]. Over the past few decades, China has witnessed significant technological advancements in the agricultural sector, facilitating the transfer of a substantial amount of rural labor and capital to non-farm industries [18].

The direct consequence of farmers’ migration from rural to urban areas, or from the agricultural sector to the non-agricultural sector, is the reallocation of surplus rural labor, resulting in an increase in rural marginal productivity [19]. However, such a significant shift may create a labor shortage in rural areas, thereby enhancing the wage-bargaining power of rural labor. Li et al. (2013) [4] proposed that the rise in wages of unskilled workers in rural China can be partly attributed to the tightening of the labor force in young age cohorts. On the other hand, Wiggins and Keats (2015) [20] argued that changes in the rural working population may be the single most powerful driver in Asian countries.

#### Agricultural mechanization and plant biotechnology

Since the 1990s, Chinese smallholder farmers have increasingly embraced agricultural mechanization to compensate for the shrinking labor force caused by the migration of rural labor to the non-agricultural sector [2, 7]. Especially after 2003, the relative price of machines against agricultural labor has experienced a significant reduction, driven by a substantial increase in real agricultural wages, while the real price of machinery has remained relatively stable [7]. As China has undergone successful industrialization, wage growth has become increasingly influential, creating pressure on farmers to replace labor with machines [2, 7]. Additionally, a phenomenon known as machinery outsourcing can be observed in China, where farmers outsource power-intensive production stages such as harvesting to specialized mechanization service providers [21]. Thus, despite the small landholdings, high land fragmentation, and predominance of older people in agricultural production, Chinese smallholder farmers can sustain their agricultural activities by adopting mechanized production methods [4, 21].

Another factor contributing to the enhanced agricultural production in China is the advancement of agricultural cultivation technology, specifically plant biotechnology. The development and application of plant biotechnology to crops such as rice, wheat, potatoes, and peanuts has led to increased productivity [3, 22, 23]. Notably, the development and widespread adoption of hybrid rice have made a substantial contribution to China’s rice yield, which rose from 1.89 t/hm2 in 1949 to 7.04 t/hm2 in 2020, significantly boosting the country’s grain production [24–26].

### Demand side

Examinations of the demand side focus on three main aspects. First is consumer demand. As individuals experience an increase in income, their consumption of agricultural products may also increase. However, owing to the Engel effect [27], which suggests that demand for non-agricultural products increases at a faster rate with rising income, the income elasticity of demand for agricultural products is lower than that for non-agricultural products [8]. China’s Engel coefficient has decreased over time, leading to a decline in the income elasticity of demand for food from 0.55 in 1978 to 0.08 in 2015 [28]. Consequently, food demand has become highly inelastic, indicating that further income increases are unlikely to significantly boost it.

Second is investment demand. Since the turn of the 21st century, the massive migration of rural labor to urban areas has led to labor shortages and increased labor costs in rural China. In response, Chinese farmers increased their investment in agricultural machinery, resulting in labor-saving measures [2, 4, 7, 21]. Additionally, the Chinese government has consistently invested resources in enhancing rural infrastructure [29–31]. The combination of private investment and government-led public investment drives technological progress and improvements in labor productivity in agricultural sector.

Third is import and export demand. With China’s accession to the World Trade Organization (WTO) in 2001 [15, 32], the import and export of agricultural products have become a significant factor affecting China’s agricultural market. International market competition resulting from trade liberalization [33] plays a role in shaping the dynamics of China’s agricultural sector and influences agricultural wages [6, 34–36].

### Industrial linkages

Agriculture exhibits strong forward and backward linkages with other industrial sectors [10, 37]. For instance, an increase in the consumption of agricultural products not only directly contributes to output, prices, and incomes within the agricultural sector but also indirectly influences the incomes of individuals involved in the upstream and downstream sectors of the agricultural production chain. Similarly, an expansion in consumption in the non-farm sector can indirectly contribute to an increase in agricultural wages through an intricate network of production linkages. In other words, according to the input-output theory, changes in final demand not only directly impact production processes and income distribution in the agricultural sector but also have indirect effects on agricultural wages through production linkage networks [37–39].

### Agricultural support policies

Since the initiation of reform and opening-up, China has implemented various agricultural policies (Table 1). Notably, China is perhaps the most prominent example of a developing country that has transitioned from taxation to agricultural support [11]. In the pre-1980s, China imposed significant taxes on agricultural production and exports [13, 40], and China’s agriculture remained taxed until the 1990s [11]. However, since the beginning of the new century, China has initiated an extensive agricultural support program encompassing tax cuts, direct subsidies, price support, policy loans, and intergovernmental transfers. In 2006, China eliminated the agricultural tax [11, 12, 41]. Furthermore, China established a procedural mechanism linking grain subsidies and price support to farmers’ increased production costs, resulting in an increase in agricultural production spending in China’s fiscal budget to $75 billion in 2012, equivalent to a subsidy of $127 per ton of grain [11].

**Table 1:** Timeline of Chinese agricultural policies.

Time	Policy measure	Types of policies
1978–1984	The household contract responsibility system with remuneration linked to output was gradually established	Market-oriented reforms
1985–1991	Contract purchasing and market purchasing of agricultural products were gradually introduced	Market-oriented reforms
1992–2002	Deepening the market-oriented reform of agriculture, which has led to the transformation from primarily relying on administrative measures for regulation and control to employing more economic and legal means	Market-oriented reforms
2000	Pilot reforms of rural taxes and fees	Tax policy
2002–2003	Soybean seed subsidy and pilot grain subsidy programs in several regions	Subsidy policy
2004	Minimum purchase price for grain	Minimum price policy
2004–2006	Direct payment to grain producers General-input subsidy Improved seed subsidy Agricultural machinery purchase subsidies Transfer payments to grain counties	Tax and subsidy policy
2006	Eliminated agricultural tax, specialty crop and animal slaughter taxes	Tax policy
2008	General-input subsidy linked to input prices Support prices for corn, soybeans, rapeseed Strategy of raising price supports annually adopted	Subsidy policy
2012	Domestic vegetable marketing sectors exempt from Value-added tax	Tax policy
2013	Targeted poverty alleviation (TPA) program	Poverty alleviation policy
2016	Direct grain subsidies, subsidies for improved seeds, and comprehensive subsidies for agricultural materials were merged into agricultural support and protection subsidies	Subsidy policy
2017	The rural revitalization strategy	Poverty alleviation policy

**Source:** Compiled based on information from [11, 41] and the Ministry of Agriculture and Rural Affairs of China.

The second significant agricultural support policy was the implementation of a minimum purchase price for grain [41]. In 2004, the Chinese government introduced a minimum purchase price program for rice, which was determined based on the cultivation costs and a reasonable profit (15‒20% of the price) and later extended to wheat and corn [42]. This policy aimed to safeguard farmers against temporary price declines. State-owned reserve corporations play a crucial role in this policy by utilizing commodity reserves as a buffer stock—purchasing commodities when market prices fall to the floor price, storing and selling them at auctions during periods of rising prices [11]. Consequently, this policy ensures a stable net return for farmers.

The third support policy focuses on eradicating poverty. Since its establishment in 1949, the People’s Republic of China has made significant efforts to address poverty [43], progressing through six stages of antipoverty efforts [44]. One notable initiative is the targeted poverty alleviation (TPA) program, which was implemented in 2013. It encompasses 10 major projects, including vocational education and training, poverty alleviation resettlement, e-commerce, and entrepreneurship training [14]. The primary goal of the TPA program was to uplift approximately 70 million people from poverty by 2020 [14, 45]. Multiple studies have indicated the effectiveness of China’s TPA program in increasing the income of impoverished rural households [14, 31, 45–48].

### IOA and SDA

IOA is widely recognized as one of the most valuable economic methods, providing a powerful tool for examining the interdependencies among industries within an economy [39, 49]. Despite the fact that the input-output model has inherent limitations, including fixed prices and unconstrained resources [50]. Within the IOA framework, SDA is a commonly employed method for quantitatively analyzing the drivers of historical changes in indicators such as output, value added, employment, industrial wages, and carbon emissions [51–59]. The fundamental concept underlying SDA is that when an economic variable can be expressed as a multiplication of various factors, it becomes possible to divide the variation in that variable into a cumulative sum of the changes in those factors. Thus, we can effectively identify the primary factors that influence the aforementioned economic variable [60].

However, despite the widespread use of this approach, there have been limited applications of IOA and SDA in analyzing the factors influencing agricultural wages. Among the few studies exploring agriculture, SDA has primarily been used to investigate the growth and drivers of environmental variables in the agricultural sector [61, 62].

From the above analysis, it is evident that the existing literature has examined agricultural wages from the perspectives of supply, demand, industrial linkages, and agricultural support policies. However, most studies have only investigated the impact of one or a few factors on agricultural wages without considering these factors within a systematic framework. In light of this gap, this paper follows the classic literature in the field of SDA [63–65] and proposes an analytical framework that combines IOA and SDA to systematically analyze how supply, demand, industrial linkages, and agricultural support policies collectively impact agricultural wages.

## 3. Methods and data

### Input-output model for measuring agricultural wages

The input-output model is a quantitative economic framework that illustrates inter-industry relationships within an economy, showing how the output of one industrial sector can become an input for another industrial sector. It serves as a valuable tool for understanding and analyzing the connections between production and consumption sectors [38, 54]. In our input-output model, we formulate the equilibrium equation for each sector of the economy as

$$\begin{array}{l} x_{1}=x_{11}+\cdots +x_{1j}+\cdots +x_{1n}+f_{1}=\sum_{j=1}^{n}x_{1j}+f_{1}\\ \vdots \;\vdots \;\vdots \;\vdots \;\vdots \;\vdots \\ x_{i}=x_{i1}+\cdots +x_{ij}+\cdots +x_{in}+f_{i}=\sum_{j=1}^{n}x_{ij}+f_{i}\\ \vdots \;\vdots \;\vdots \;\vdots \;\vdots \;\vdots \\ x_{n}=x_{n1}+\cdots +x_{nj}+\cdots +x_{nn}+f_{n}=\sum_{j=1}^{n}x_{nj}+f_{n} \end{array}$$ (1)

where *x*_*i*_ represents the total output of sector *i*, *x*_*ij*_ denotes inter-industry sales from sector *i* to sector *j*, and *f*_*i*_ denotes the final demand of sector *i*. By defining the direct input coefficients *a*_*ij*_ = *x*_*ij*_ / *x*_*i*_, Eq (1) can be represented in matrix form as

X=AX+F (2)

where $X={\left[\begin{array}{cccc} x_{1} & x_{2} & \cdots & x_{n} \end{array}\right]}^{\prime }$, $A={\left[a_{ij}\right]}_{n\times n}$, and $F={\left[\begin{array}{cccc} f_{1} & f_{2} & \cdots & f_{n} \end{array}\right]}^{\prime }$. Eq (2) is obtained through transformation as

$$X={\left(I-A\right)}^{-1}F=(I+A+A^{2}+\cdots +A^{k})F=BF$$ (3)

Here, *I* represents the unit matrix, and *B* = (*I—A*)^-1^ = (*I* + *A* + *A*^2^ +… + *A*^*k*^) (*k* = ∞) represents the Leontief inverse matrix. The economic implication of this indicator is that final demand initiates the production of the final products and triggers a series of supply and demand for intermediate products, denoted as *AF*, *A*^*2*^*F*,… *A*^*k*^*F*. Consequently, the production activities required to meet final demand extend beyond the production of the final good. This indicator provides valuable insights into the direct and indirect inputs required throughout the production chain for the final good, thereby highlighting the interdependencies among different industrial sectors [39, 50, 66, 67].

In China’s input-output tables, value-added includes compensation of employees, net taxes on production, depreciation of fixed capital and operating surplus. Let *w* be the vector of compensation of employees, and *l* be the vector of employed persons. The average wage of employed persons in each sector can then be calculated as

$$\omega =\frac{w}{1}$$ (4)

To establish the relationship between average wage, industrial linkages, final demand, and agricultural support policies within an input-output framework, we can reformulate the equation as follows:

$$\omega =\frac{1}{l}\times \frac{w+t}{X}\times \frac{w}{w+t}\times BF$$ (5)

In Eq (5), *t* represents the vector of net taxes on production and *w*+*t* represents the pre-tax income vector. The term (*w*+*t*)/*X* indicates the share of pre-tax income in total sectoral output. Denoting *T* as *w*/(*w*+*t*), we can infer that if *T* = *w*/(*w*+*t*) < 1, the government taxes productive income, and if *T* = *w*/(*w*+*t*) > 1, subsidies are provided to employed persons. Let *L* be the diagonal matrix of 1/*l*, *R* be the diagonal matrix of (*w*+*t*)/*X*, and *T* be the diagonal matrix of *w*/(*w*+*t*). Eq (6) was obtained as follows:

ω=L×R×T×B×F (6)

From Eq (6), it is evident that average wage of employed persons is influenced by various factors, including the labor matrix *L*, pre-tax income share matrix *R*, tax moderation matrix *T*, Leontief inverse matrix *B*, and final demand matrix *F*.

### SDA for factors affecting the growth of agricultural wages

**Fig 1:**
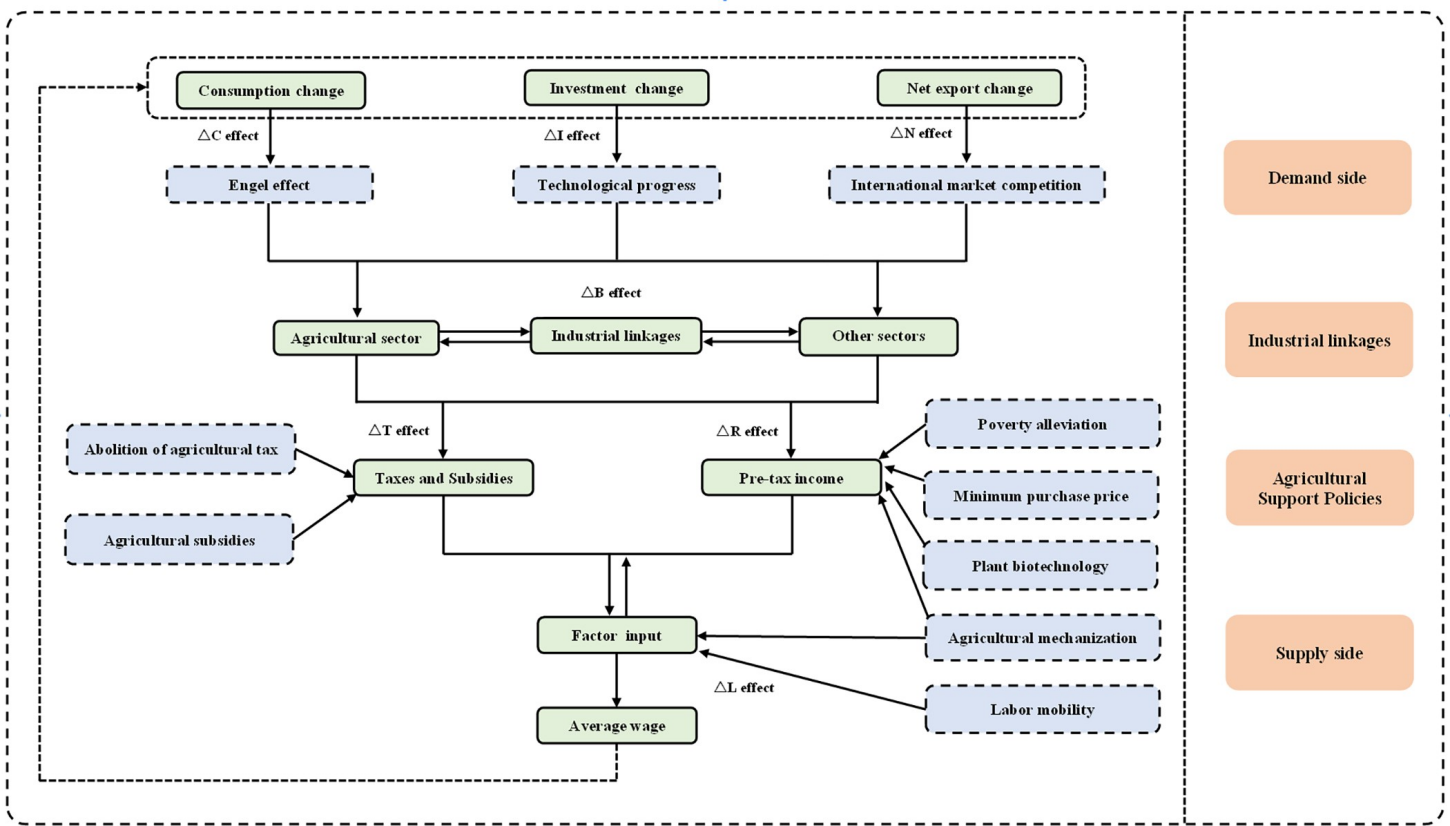
Factors affecting the growth of Chinese agricultural wages.

The SDA technique is widely employed within an input-output framework to quantitatively identify the underlying drivers of changes in economic indicators over time [50, 65]. In this study, we used SDA to analyze the factors influencing the growth of agricultural wages, considering four dimensions: supply, demand, industrial linkages, and agricultural support policies (Fig 1).

Starting on the supply side, changes in labor migration, agricultural mechanization, and advancements in plant biotechnology have significant impacts on agricultural wages. The transfer of surplus rural labor and increased agricultural mechanization contribute to higher marginal productivity in rural areas [19]. Additionally, the migration of rural labor to urban or non-agricultural sectors might tighten rural labor markets, thereby increasing the bargaining power of rural labor [4]. Moreover, improvements in agricultural mechanization and plant biotechnology can enhance crop productivity and yield [3, 22, 23].

On the demand side, changes in consumption demand, investment demand, and import/export demand can affect agricultural wages. Fluctuations in consumption levels significantly influence income from the sale of agricultural products. However, it is important to consider the Engel effect, which suggests that as real income increases, the income elasticity of agricultural demand tends to decrease [28, 37]. Investment changes influence agricultural wages through technological advancements and enhanced labor productivity [2, 7, 29, 31]. Furthermore, shifts in import and export volumes affect agricultural markets through international market competition [6, 33, 34, 36].

Changes in industrial linkages resulting from technological advancements, factor substitution, and shifts in output composition can directly or indirectly influence agricultural wages [39, 63, 65]. Notably, alterations in the forward and backward linkages connecting agriculture with other sectors can indirectly influence agricultural wages through upstream or downstream production connections [10, 37–39].

Regarding agricultural support policies, changes in taxes, subsidies, minimum purchase prices, and anti-poverty initiatives can directly or indirectly influence agricultural wages. Taxes and subsidies directly affect agricultural wages by reducing or increasing it. The minimum purchase price policy safeguards farmers from declining agricultural prices and ensures stable net returns [11]. Anti-poverty initiatives affect agricultural wages through various channels, such as job creation, increased agricultural sales, and skill enhancement [14, 44].

Based on the comprehensive analysis conducted above, we decomposed the factors contributing to the growth in agricultural wages. Employing the decomposition approach proposed by Dietzenbacher and Los (1998) [65], we can express the changes in sectoral average wage between the two periods (*ω*_1_-*ω*_0_) as follows:

$$\begin{array}{l} \Delta \omega =\omega_{1}-\omega_{0}=L_{1}R_{1}T_{1}B_{1}F_{1}-L_{0}R_{0}T_{0}B_{0}F_{0}\\ =\Delta LR_{0}T_{0}B_{0}F_{0}+L_{1}\Delta RT_{0}B_{0}F_{0}+L_{1}R_{1}\Delta TB_{0}F_{0}+L_{1}R_{1}T_{1}\Delta BF_{0}+L_{1}R_{1}T_{1}B_{1}\Delta F\\ =\Delta LR_{1}T_{1}B_{1}F_{1}+L_{0}\Delta RT_{1}B_{1}F_{1}+L_{0}R_{0}\Delta TB_{1}F_{1}+L_{0}R_{0}T_{0}\Delta BF_{1}+L_{0}R_{0}T_{0}B_{0}\Delta F\\ =\underset{\Delta L\;effect}{\underset{︸}{\frac{1}{2}(\Delta LR_{0}T_{0}B_{0}F_{0}+\Delta LR_{1}T_{1}B_{1}F_{1})}}+\underset{\Delta R\;effect}{\underset{︸}{\frac{1}{2}(L_{1}\Delta RT_{0}B_{0}F_{0}+L_{0}\Delta RT_{1}B_{1}F_{1})}}\\ +\underset{\Delta T\;effect}{\underset{︸}{\frac{1}{2}(L_{1}R_{1}\Delta TB_{0}F_{0}+L_{0}R_{0}\Delta TB_{1}F_{1})}}+\underset{\Delta B\;effect}{\underset{︸}{\frac{1}{2}(L_{1}R_{1}T_{1}\Delta BF_{0}+L_{0}R_{0}T_{0}\Delta BF_{1})}}\\ +\underset{\Delta C\;effect}{\underset{︸}{\frac{1}{2}(L_{1}R_{1}T_{1}B_{1}\Delta C+L_{0}R_{0}T_{0}B_{0}\Delta C)}}+\underset{\Delta I\;effect}{\underset{︸}{\frac{1}{2}(L_{1}R_{1}T_{1}B_{1}\Delta I+L_{0}R_{0}T_{0}B_{0}\Delta I)}}\\ +\underset{\Delta N\;effect}{\underset{︸}{\frac{1}{2}(L_{1}R_{1}T_{1}B_{1}\Delta N+L_{0}R_{0}T_{0}B_{0}\Delta N)}}+\frac{1}{2}(L_{1}R_{1}T_{1}B_{1}\Delta O+L_{0}R_{0}T_{0}B_{0}\Delta O) \end{array}$$ (7)

The first term on the right-hand side of Eq (7) represents the change in the labor input coefficient, reflecting the impact of mobility in agricultural labor on agricultural wages. This effect is referred to as the labor mobility effect (Δ*L* effect). The second term captures the influence of changes in the share of pre-tax income in the total output of the agricultural sector on agricultural wages, referred to as the pre-tax income share effect (Δ*R* effect). The third term signifies the effect of variations in government tax policies on agricultural wages, referred to as the tax effect (Δ*T* effect). The fourth term indicates the impact of changes in industrial linkages on agricultural wages, denoted as the industrial linkage effect (Δ*B* effect). Finally, the remaining terms represent the effect of changes in final demand on agricultural wages, known as the final demand effect (*ΔF* effect).

In the published input-output tables for China, final demand can be further categorized as *F* = *C*+*I*+*E*-*M*+*O*, where *C* represents consumption expenditure, *I* represents gross capital formation, *E* represents exports, *M* represents imports, and *O* represents other factors. Let *N* be net exports (*E*—*M*). Therefore, the final demand effect can be further subdivided into three components: the consumption effect (Δ*C* effect), which reflects the impact of changes in consumption demand on agricultural wages; the investment effect (Δ*I* effect), which represents the influence of changes in investment demand on agricultural wages; and the net export effect (Δ*N* effect), which represents the effect of changes in net exports on agricultural wages.

### Data

This study utilized competitive input-output tables spanning 1981 to 2020. The input-output tables for the years 1981 to 2018 were sourced from Zhang et al. (2021) [68]. These tables were constructed by the School of Applied Economics at Renmin University of China, utilizing both the China input-output tables and the input-output extension table compiled by the National Bureau of Statistics of China (NBSC).Zhang et al. (2021) [68] employed statistical information and mathematical methods such as the RAS method, GRAS method, and mathematical programming to compile sequential input-output tables covering 1981 to 2018. Building on this methodology, this study adjusted and aligned with the 2020 competitive input-output table published by the NBSC. This study did not utilize the 2019 input-output table for calculations; therefore, the 2019 input-output table was not computed. Regarding agricultural wages in 2019, we employed the average wage of employed persons in the agricultural sector, as published by the NBSC.

The input-output tables used in this study originally included 18 industrial sectors. To focus on agricultural wages, this study simplified the analysis by aggregating these 18 sectors into three major industrial sectors, according to three industries classification regulations enacted in 2018 by the NBSC. The categorizations are shown in Table 2.

**Table 2:** Industrial sector classification.

Three industries	Industrial sectors
Agriculture	Farming, Forestry, Animal Husbandry and Fishery
Manufacturing	Mining and Quarrying
	Food and Tobacco
	Textile, Garments, Leather, Down and Related Products
	Timber Processing, Furniture Manufacturing, Papermaking and Paper Products, Cultural, Educational, and Sports Articles
	Petroleum, Coking and Chemical Products
	Nonmetal Mineral Products
	Metal Products
	Machinery Equipment, Transportation Equipment, Electronic and Telecommunications Equipment
	Other Manufacturing Industry
	Production and Supply of Electricity, Gas and Water
	Construction
Services	Wholesale and Retail
	Transport, Storage and Post
	Information Transmission, Software and Information Technology Services
	Finance and Real Estate
	Research and Development
	Other Services

To ensure comparability of the data across different years, the input-output tables in current prices were adjusted to constant 2010 prices using the producer price index for industrial products obtained from the NBSC. This is guided by the fact that the input-output table predominantly centers around production activities, and the producer price index for industrial products typically offers a more accurate reflection of price changes in the production process. The data of employed persons were sourced from the China Labor Statistical Yearbook.

## 4. Results

### Trend of Chinese agricultural wages from 1981 to 2020

**Fig 2:**
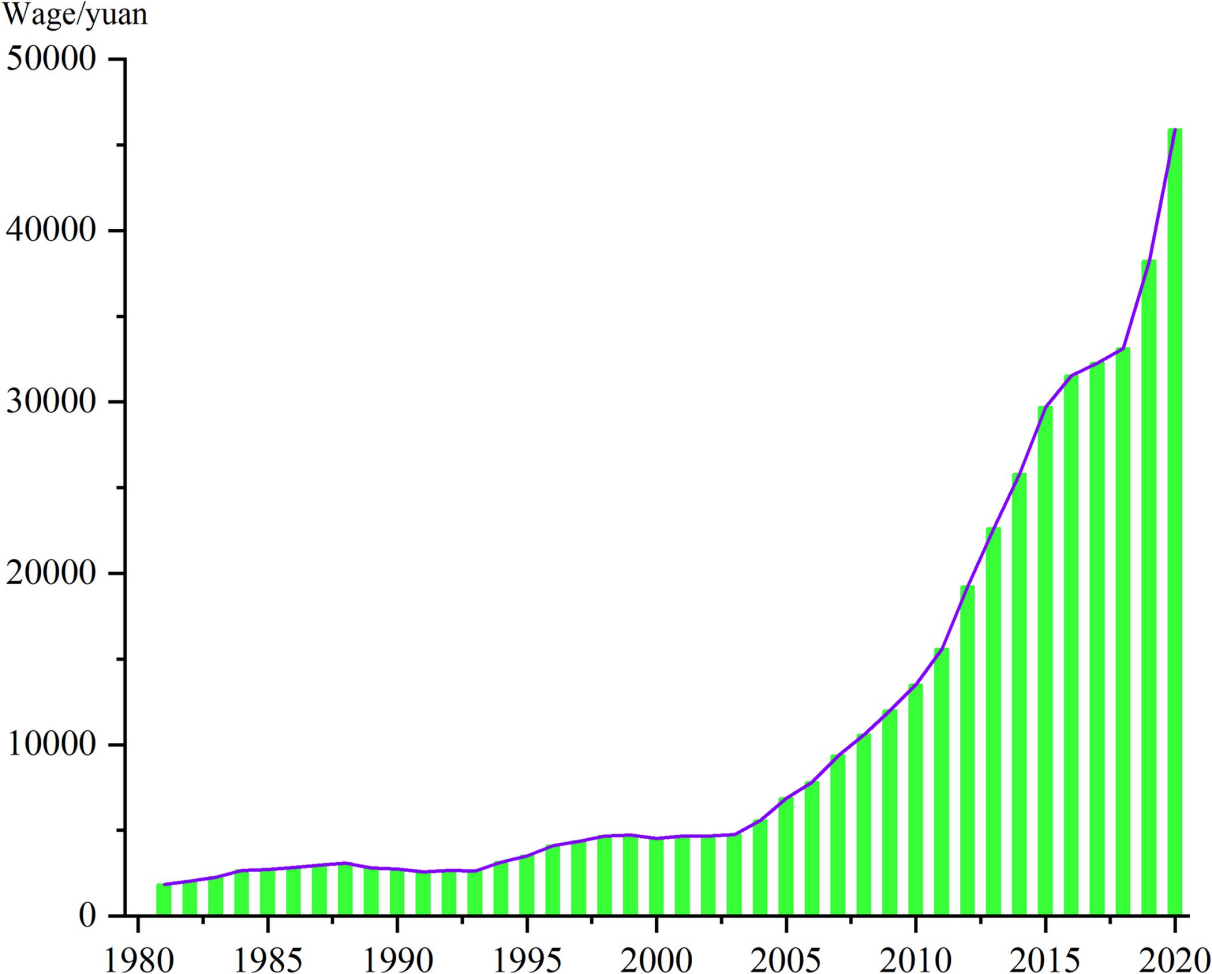
Trend of Chinese agricultural wages during 1981‒2020 (yuan at constant 2010 prices).

Fig 2 illustrates the remarkable upward trajectory of Chinese agricultural wages from 1981 to 2020, culminating at 45,901.19 yuan in 2020. This signifies a notable increase of 24.74 times compared to the wage level observed in 1981, with an average annual growth rate of 8.57%.

An in-depth analysis of the distinct time intervals revealed discernible patterns. During the period from 1981 to 1990, Chinese agricultural wages experienced a growth rate of 1.48 times, rising from 1,855.70 yuan to 2,742.54 yuan. Subsequently, from 1990 to 2000, their wage demonstrated a growth rate of 1.65 times, increasing from 2,742.54 yuan to 4,538.29 yuan. Notably, the period between 2000 and 2010 witnessed a substantial growth rate of 2.97 times, as Chinese agricultural wages surged from 4,538.29 yuan to 13,495.16 yuan. Lastly, in the timeframe spanning 2010 to 2020, Chinese agricultural wages experienced an exceptional growth rate of 3.40 times, soaring from 13,495.16 yuan to 45,901.19 yuan. The accelerated growth trend observed in Chinese agricultural wages after 2000 is particularly noteworthy.

### Decomposition results

The decomposition results revealed that the leading factors driving changes in agricultural wages varied across different periods (Table 3). During the period from 1981 to 1990, Chinese agricultural wages witnessed an increase of 886.84 yuan, primarily propelled by the consumption effect (133.24%), investment effect (52.19%), and net export effect (9.64%). However, these positive drivers were partly offset by a decline in the labor mobility effect (-72.01%), pre-tax income share effect (-11.05%), and industrial linkage effect (-9.98%). In the period from 1990 to 2000, Chinese agricultural wages experienced an increase of 1795.75 yuan, mainly driven by the consumption effect (107.84%), investment effect (31.13%), and labor mobility effect (15.41%). Nonetheless, the positive impact of these factors was somewhat mitigated by a decline in the industrial linkage effect (-40.96%) and the pre-tax income share effect (-27.93%). Notably, for both the periods 2000‒2010 and 2010‒2020, the industrial linkage effect and net export effect emerged as the factor contributing to a decline in agricultural wages, whereas the other factors contributed positively to wage growth.

**Table 3:** Decomposition results on the growth of agricultural wages in China during 1981‒2020.

Time	Δ*ω*	Δ*L*	Δ*R*	Δ*T*	ΔB	ΔC	ΔI	ΔN	ΔO
1981‒1990	886.84 (100.0%)	-638.64 (-72.01%)	-98.03 (-11.05%)	5.12 (0.58%)	-88.53 (-9.98%)	1181.59 (133.24%)	462.81 (52.19%)	85.53 (9.64%)	-23.03 (-2.60%)
1990‒2000	1795.75 (100.0%)	276.70 (15.41%)	-501.54 (-27.93%)	-0.62 (-0.03%)	-735.49 (-40.96%)	1936.55 (107.84%)	558.94 (31.13%)	-34.19 (-1.90%)	295.39 (16.45%)
2000‒2010	8956.87 (100.0%)	2177.69 (24.31%)	500.87 (5.59%)	227.19 (2.54%)	-427.64 (-4.77%)	2003.12 (22.36%)	4742.64 (52.95%)	-358.52 (-4.00%)	91.53 (1.02%)
2010‒2020	32406.03 (100.0%)	12285.19 (37.91%)	1100.78 (3.40%)	1475.59 (4.55%)	-4681.79 (-14.45%)	13872.81 (42.81%)	9113.00 (28.12%)	-162.99 (-0.50%)	-596.56 (-1.84%)
1981‒2020	44045.49 (100.0%)	9928.54 (22.54%)	-1112.07 (-2.52%)	1387.95 (3.15%)	-13818.80 (-31.37%)	26380.85 (59.89%)	21702.38 (49.27%)	-483.93 (-1.10%)	60.57 (0.14%)

**Note:** The figures in parentheses represent percentage changes in agricultural wages throughout the analysis period.

Overall, the factors that significantly contributed to the increase in agricultural wages throughout the entire period of 1981‒2020 were the consumption effect (59.89%), investment effect (49.27%), labor mobility effect (22.54%), and tax effect (3.15%). Conversely, the factors that led to a decrease in agricultural wages were the industrial linkage effect (-31.37%), pre-tax income share effect (-2.52%), and net export effect (-1.10%).

#### Labor mobility effect (ΔL)

**Fig 3:**
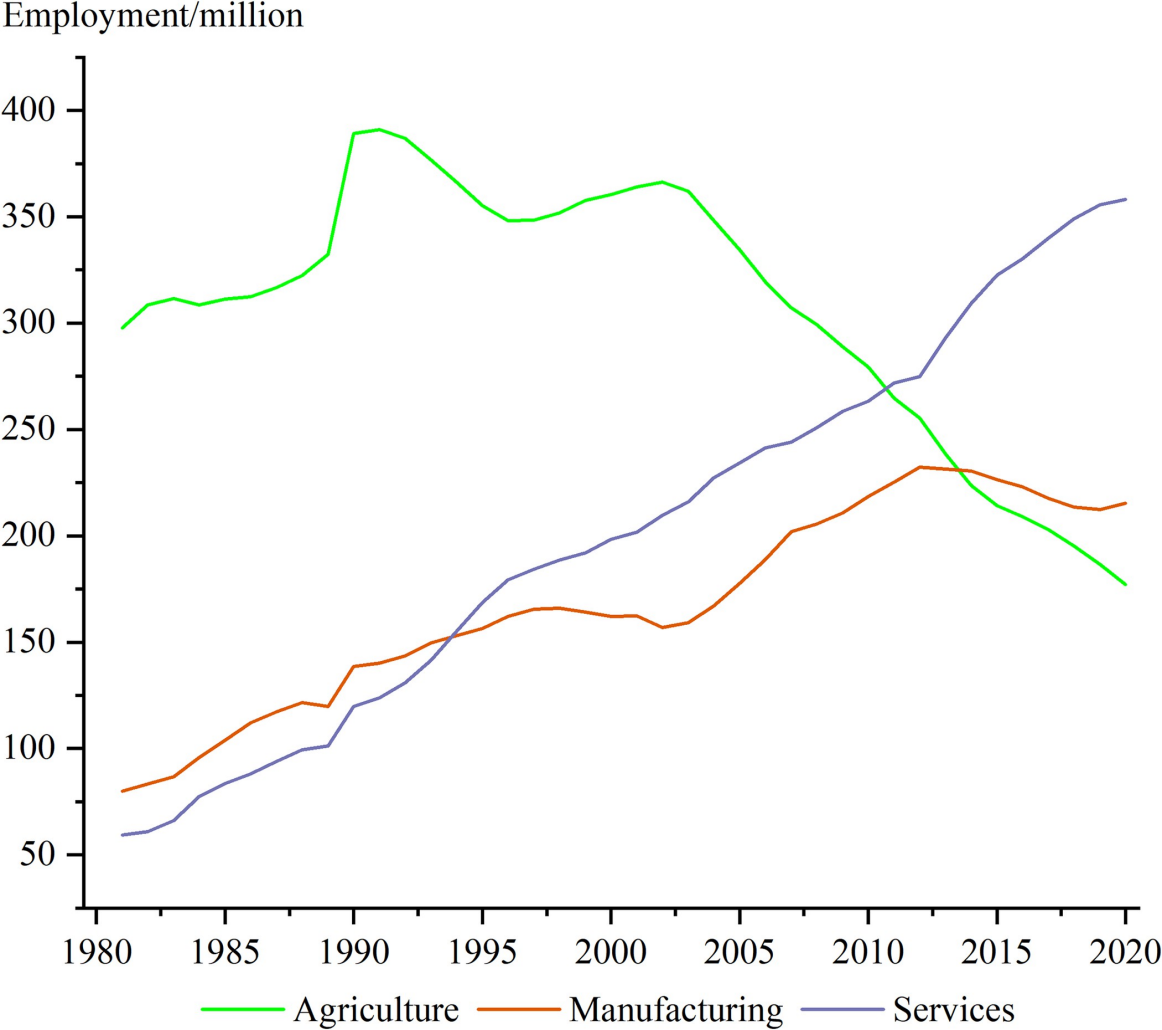
Changes in employed persons by three industries in China during 1981‒2020.

As presented in Table 3, the labor mobility effect exhibited a negative impact on agricultural wages during the period of 1981‒1990, but a positive impact in subsequent time periods. Several factors may account for this observation. First, during the years 1981‒1990, China witnessed a substantial increase in its labor force. As the country was in the early stages of implementing its reform and opening up, the market economy system was not fully established [28]. The non-agricultural sector experienced slow development, leading to limited absorptive capacity for the significant influx of labor. Additionally, strict immigration controls were in effect at that time [5, 16], resulting in the concentration of the working population in rural areas and the emergence of a surplus labor force. Fig 3 illustrates the continuous growth in the number of employed persons in the agricultural sector from 297.77 million in 1981 to 389.14 million in 1990.

However, in subsequent years, with the deepening of market-oriented reforms [28], the industrial and service sectors underwent a significant expansion. Moreover, restrictions on rural migration in China have eased [5, 16, 17]. Consequently, a portion of the agricultural labor force transitioned to the industrial and service sectors. Particularly after 2000, gradual implementation of market mechanisms and China’s accession to the WTO [28, 69] facilitated the accelerated growth of the industrial and service sectors, absorbing a considerable number of rural labor. Consequently, the number of employed persons in the agricultural sector noticeably declined after 2000.

Based on the decomposition results of the labor mobility effect, we conclude that rural labor transfers were beneficial for releasing surplus agricultural labor [19] and improving labor productivity in the agricultural sector. Simultaneously, rural labor transfer influenced the supply and demand dynamics in the rural labor market [4], thereby enhancing the bargaining power of rural labor. Both aspects effectively contributed to an increase in agricultural wages. This also suggests that with the growth of agricultural mechanization in China [2, 7, 21], the productivity of the agricultural sector has significantly improved, enabling higher agricultural output with reduced labor input. In terms of contribution, the labor mobility effect accounted for 22.54% of the increase in agricultural wages from 1981 to 2020, ranking third in terms of contribution.

#### Pre-tax income share effect (ΔR)

As observed in Table 3, the pre-tax income share effect had a negative influence on agricultural wages during the period spanning 1981‒2000, but exhibited a positive impact during 2000‒2020.

The negative pre-tax income share effect observed between 1981‒2000 can be attributed to a combination of factors. Despite the increase in agricultural production during this period, low food prices prevailed, leading to a slight deterioration in farmers’ welfare [28]. It was not until 2004 that China implemented a minimum purchase price policy for grain, ensuring that grain prices remained high [11, 42]. Consequently, there are three primary reasons for the positive pre-tax income share effect witnessed in 2000‒2020. First, agricultural mechanization and the adoption of plant biotechnology significantly enhanced agricultural production [2, 3, 7, 18, 24, 26]. Second, the minimum purchase price policy enforced by the Chinese government ensured the stability of agricultural product prices, thereby fostering wage growth among farmers.

Additionally, the further increase in the pre-tax income share effect during 2010‒2020 can be attributed to the effective implementation of China’s targeted poverty alleviation program, which commenced in 2013. This policy successfully facilitated poverty alleviation efforts and promoted industrial development in impoverished regions, thereby augmenting agricultural wages growth [14, 31, 45, 47].

#### Tax effect (ΔT)

Tax effect had a limited impact on increasing agricultural wages during the periods 1981‒1990 and 1990‒2000. This can be attributed to the taxation of agriculture and the relatively low government support provided to the agricultural sector in 1981‒2000 [11, 40]. However, in the periods 2000‒2010 and 2010‒2020, the tax effect exhibited a significant positive impact, contributing 2.54% and 4.55%, respectively. Table 4 illustrates the variations in the tax moderation coefficient in China’s agricultural sector, indicating an increase from 97.01% to 99.80% during 2000‒2010, and a further increase from 99.80% to 105.87% during 2010‒2020.

**Table 4:** Tax moderation coefficients in Chinese agricultural sector.

	1981‒1990	1990‒2000	2000‒2010	2010‒2020	1981‒2020
*T*_0_	96.83%	97.02%	97.01%	99.80%	96.83%
*T*_1_	97.02%	97.01%	99.80%	105.87%	105.87%
△*T*	0.20%	-0.02%	2.79%	6.07%	9.04%

This notable shift can be attributed to the implementation of China’s rural tax reform, which commenced in 2000 and has gradually expanded in scope. Notably, the complete abolition of agricultural tax began in 2006 [11, 12], and subsequently, in 2012, there was a significant increase in subsidies allocated to agriculture [11]. These tax support policies effectively reduced the burden on farmers and improved their wage.

#### Industrial linkage effect (ΔB)

Table 3 demonstrates that the industrial linkage effect had the most substantial negative impact on agricultural wages growth during the period 1981‒2020, with a contribution of -31.37%.

**Fig 4:**
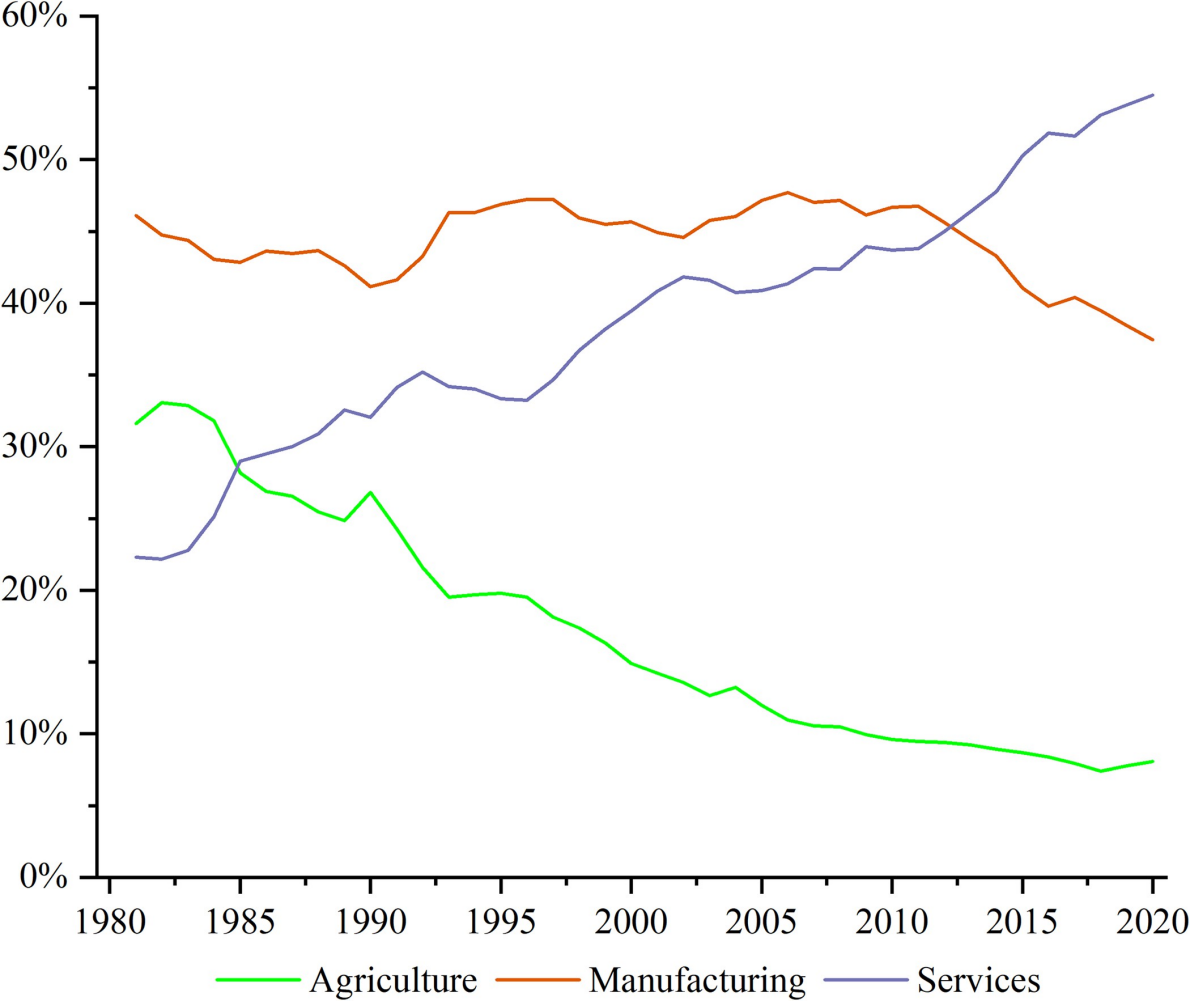
Changes in the share of three industries in China during 1981‒2020.

Table 5 presents the changes in the Leontief inverse matrices for three industries in China. The sum of the cross-sectional sums of the agricultural sector was negative in each period, indicating a gradual decline in the share of agriculture in the national economy’s production chain. Considering that agricultural products are primarily used for food consumption and industrial raw materials, the main reason for the decline in the agricultural sector’s share in the national economy’s production chain could be attributed to Kuznets-type industrial structural changes (i.e., the declining share of agriculture in the national economy as it grows, as illustrated in Fig 4). As the share of agriculture in China’s national economy decreases, the relative importance of agriculture in the division of labor within the production chain of the national economy also diminishes. Consequently, other sectors reduce their direct or indirect inputs into agriculture during the production process, negatively impacting agricultural wages.

**Table 5:** Changes in the Leontief inverse matrix for three industries in China.

	Industries	Agriculture	Manufacturing	Services	Total
1981‒1990	Agriculture	0.06	-0.02	-0.06	-0.02
	Manufacturing	0.02	0.07	-0.21	-0.12
	Services	0.03	0.06	0.04	0.13
1990‒2000	Agriculture	-0.04	-0.10	-0.01	-0.15
	Manufacturing	0.30	0.31	0.24	0.85
	Services	0.11	0.12	0.09	0.31
2000‒2010	Agriculture	-0.02	0.01	-0.03	-0.05
	Manufacturing	0.17	0.53	0.03	0.73
	Services	-0.02	-0.01	-0.07	-0.09
2010‒2020	Agriculture	-0.01	-0.03	-0.02	-0.06
	Manufacturing	-0.28	-0.58	-0.32	-1.18
	Services	0.04	0.16	0.21	0.40
1981‒2020	Agriculture	-0.01	-0.14	-0.13	-0.29
	Manufacturing	0.21	0.33	-0.26	0.28
	Services	0.16	0.33	0.27	0.75

**Note:** The horizontal summation of individual rows in the Leontief inverse matrix denotes the magnitude of total output increment required across each sector of the national economy in response to a one-unit increase in final demand within that specific sector.

#### Final demand effect (ΔF)

The final demand effect (Δ*F*) emerged as the dominant driver of agricultural wages growth, making a substantial cumulative contribution of 108.21% over the period 1981‒2020. Remarkably, the consumption effect (Δ*C*) surpassed the investment effect (Δ*I*) in all time periods except 2000‒2010. This phenomenon can be attributed to the investment boom witnessed in China during the nearly 10 years following its accession to the WTO, particularly in rural infrastructure construction and agricultural machinery [7, 30]. As a result, the investment effect played a more prominent role than the consumption effect in driving agricultural wages growth during the 2000‒2010 period.

Regarding the consumption effect (Δ*C*), its contribution to agricultural wages growth generally demonstrated a declining trend, substantiating the presence of the Engel effect (The diminished significance of the consumption effect from 2000 to 2010 can be considered an extraordinary phenomenon. This phenomenon can be attributed to the substantial investment boom in China that occurred between 2000 and 2010. During this period, there was a heightened emphasis on investment, which overshadowed the role of the consumption effect.). This effect suggests that, as individuals’ income levels rise, the proportion of income spent on agricultural products decreases [8, 27, 28]. Nonetheless, even in the presence of the Engel effect, consumer demand remained the most crucial factor influencing agricultural wages growth, ranking first in terms of contribution.

The net export effect (Δ*N*) exerted a negative impact in all periods except 1981‒1990. This outcome primarily stems from China’s status as a populous country with a significant reliance on overseas food imports. Consequently, China’s agricultural sector experienced an extended period of negative net exports. Another contributing factor is that China significantly reduced tariffs on agricultural products (except for rice, wheat, and corn) after its accession to the WTO in 2001[11]. At the same time, domestic prices for major Chinese agricultural products tend to be higher than international prices, thereby stimulating more agricultural imports [11, 13].

## 5. Discussion

When comparing the periods 1981‒2000 and 2000‒2020, a notable disparity in the growth rate of Chinese agricultural wages becomes apparent. The primary reason for this contrast is the fact that while market-oriented reforms were actively promoted in China during 1981‒2000, the establishment of a fully developed market economy system was not achieved [28]. Consequently, resource allocation efficiency remained low, leading to a slower rate of economic growth. However, after 2000, China witnessed significant economic growth, propelled by the deepening of market-oriented reforms and the positive effects of its accession to the WTO [15, 28, 69]. The liberalization of domestic markets and trade policies further reduced distortions in resource allocation [13, 40]. During this period, the substantial transfer of surplus agricultural labor to the non-agricultural sector, and the advancement of agricultural mechanization and plant biotechnology, increased final demand for agricultural products, and the implementation of agricultural support policies by the Chinese government collectively contributed to the rapid growth of agricultural wages.

Although some scholars argue that the transition of the rural labor force from the agricultural sector to the non-agricultural sector was largely completed by 2011 [4], it is crucial to recognize that the transfer of China’s agricultural labor force continues. The key reason for this continuous transition is that wages in the non-agricultural sector significantly surpass those in the agricultural sector, which serves as a magnet attracting the rural labor force to seek higher-paying employment opportunities in the non-agricultural sector [20]. Furthermore, increased mechanization in Chinese agriculture has enhanced production efficiency, enabling rural areas to engage in agricultural activities with a reduced labor force. Nonetheless, it is crucial to acknowledge that, with the aging of China’s rural population, addressing the challenges posed by labor shortages in rural areas and the high production costs resulting from land fragmentation will persist as crucial issues that China needs to tackle in the foreseeable future.

There are distinct variations in the mechanisms underlying the pre-tax income share effect between the agricultural and non-agricultural sectors. In the non-agricultural sector, capital substitution and union protection play pivotal roles in shaping the dynamics of income distribution [50, 70, 71]. Conversely, the agricultural sector is influenced by two key factors: (1) agricultural mechanization and plant biotechnology for enhanced yields [3, 18, 21, 22], and (2) the implementation of the minimum purchase price policy to stabilize agricultural product prices [11]. The fundamental distinction lies in the divergent employment patterns observed: the non-agricultural sector employs workers to produce goods or services, whereas in China’s agricultural sector, farmers simultaneously serve as both employers and laborers. Although some individuals in rural areas generate income through self-employment activities, the enterprises associated with these self-employed individuals can be characterized as microenterprises, typically employing less than 1.5 individuals per enterprise, including the entrepreneurs themselves [4]. This distinction has two implications. First, non-agricultural firms, driven by profit maximization, tend to substitute labor with cost-effective machinery. Conversely, farmers in the Chinese agricultural sector invest in machinery to alleviate their labor burden and enhance productivity, thereby enabling mechanization to compensate for the reduced rural labor [20]. Second, while the non-agricultural sector commonly features labor unions, such a mechanism does not exist in the agricultural sector. Nevertheless, it is pertinent to highlight that the Chinese government has instituted a minimum purchase price policy for agricultural products [11]. This policy aims to ensure that the benefits of farmers’ food production surpass the associated costs, thereby indirectly safeguarding their interests.

The existing literature suggests that agricultural support policies may introduce distortions in labor incentives and agricultural markets [13, 42, 72]. However, an examination of China’s agricultural support policies reveals that these policies, including tax and subsidy policies, minimum purchase price policies, and poverty alleviation programs, directly or indirectly contribute to growth in agricultural wages. A potential explanation for this phenomenon may be the Chinese government’s prioritization of three key factors: modernizing agriculture by inducing the adoption of modern inputs, reducing the income disparity between rural and urban areas, and ensuring food security [11].

Regarding the industrial linkage effect, despite the long-standing recognition of strong forward and backward linkages between agriculture and other industrial sectors [10, 37], the impact of these linkages on agricultural wages has received limited attention in the literature. Existing studies primarily rely on econometric models that do not provide a comprehensive analysis of such intersectoral linkages, treating the network of production linkages between agriculture and other sectors as an enigmatic “black box.” In China, we can observe a diminishing relative significance of agriculture within the national economy’s division of labor as the country’s economy expands. Consequently, other sectors progressively reduce their direct or indirect inputs to agriculture during the production process, thereby negatively influencing agricultural wages. Notably, among the seven factors examined in this study, the industrial linkage effect emerged as the most substantial source of the negative impact on agricultural wages growth, underscoring its indispensable role that cannot be disregarded.

## 6. Conclusions

In this study, we investigated the driving factors influencing agricultural wages growth in China. To accomplish this objective, we adopted a comprehensive approach that integrated the IOA and SDA methods. By employing this methodology, we decomposed the factors shaping agricultural wages into seven dimensions: the labor mobility effect, pre-tax income share effect, tax effect, industrial linkage effect, consumption effect, investment effect, and net export effect. They collectively represent aspects related to supply, demand, industrial linkages, and agricultural support policies.

Our findings revealed that changes in consumption demand, investment demand, and labor mobility are the primary drivers of agricultural wages growth in China. Conversely, changes in industrial linkages negatively affected agricultural wages growth. Another noteworthy observation is the transition of the pre-tax income share effect and the tax effect from a negative effect during the 1981‒2000 period to a positive effect during the 2000‒2020 period. This suggests that the agricultural support policies implemented in China since the beginning of the 21st century have contributed to the growth of agricultural wages to some extent.

Although China has indeed achieved remarkable success in elevating agricultural wages, it is imperative to acknowledge that persistent labor shortages in rural areas and high production costs resulting from extensive land fragmentation remain pivotal challenges confronting the Chinese agricultural sector. This not only influences agricultural wages but also exerts a substantial impact on China’s agricultural security. Therefore, the Chinese government should undertake the following measures: (1) Enhance subsidies for agricultural machinery and intensify efforts to advance the level of agricultural mechanization. (2) Strengthen research and development in agricultural planting technology, actively incorporate international advanced agricultural technology, and concurrently enhance farmers’ planting skills through targeted training programs. This multifaceted approach could ameliorate agricultural productivity and overall output. (3) Optimize the land transfer system and advocate for the widespread adoption of large-scale management practices for agricultural land.

The methodology employed in this study has notable strengths. It encompassed a comprehensive framework that integrated supply, demand, industry linkages, and agricultural support policies, ensuring a holistic analysis rather than a limited perspective. A pertinent example is the analysis conducted by Wiggins and Keats (2015) [20], who explored the determinants of rural wages solely from supply and industry-linked perspectives. Their study suggested that changes in the rural labor force may be the predominant driver of rural wages. In contrast, our research revealed that labor supply ranks as the third most influential factor, following consumption demand and investment demand. This disparity underscores the importance of considering multiple factors and avoiding oversimplification when understanding agricultural wages dynamics.

It is important to acknowledge the limitations of this study. First, we did not differentiate between domestic and imported products in the intermediate and final uses. Subsequent research endeavors could address this aspect by incorporating noncompetitive input-output tables. Second, the input-output model had inherent limitations, including its inability to capture feedback effects, fixed prices, and unconstrained resources, as emphasized by Shu et al. (2022) [50]. To address these limitations, future studies should explore the use of computable general equilibrium (CGE) models.

## Supporting information

S1 File (XLSX)

S2 File (XLSX)
